# What It Means to Become a Father

**DOI:** 10.1177/15579883251323251

**Published:** 2025-03-27

**Authors:** Åsa Leanderz, Maria Henricson, Frida Lygnegård, Caroline Bäckström, Margaretha Larsson

**Affiliations:** 1 School of Health Sciences, University of Skövde, Skövde, Sweden; 2 FamCeH, School of Health Sciences, University of Skövde, Skövde, Sweden; 3 Department of Caring Science, University of Borås, Borås, Sweden; 4 Jönköping Academy, School of Health and Welfare, Jönköping University, Jönköping, Sweden; 5 Department of Rehabilitation, School of Health and Welfare, Jönköping University, Jönköping, Sweden

**Keywords:** men, parent, pregnancy, phenomenological hermeneutic, transition, well-being

## Abstract

For fathers, the transition to parenthood can be experienced as an emotional phase. Fathers often state feeling overlooked and unsupported during their transition to parenthood. This study addressed this issue by exploring what it means to become a father—a qualitative design with a phenomenological hermeneutical approach. Data were collected through open-ended interviews with 19 fathers living in Sweden. The participants were encouraged to reflect on the meaning of becoming a father. Becoming a father means feeling connectedness to their child, their partner, and their friends, as well as creating strategies entailing flexibility, engagement, management, support, and solitude in their new situation. Fathers use digital media for support to create strategies, but it can evoke anxiety. The meaning of becoming a father concludes that they are deeply affected by the new situation. To support fathers during their transition to parenthood, midwives and child healthcare nurses should facilitate reflective conversations with them about their experiences of becoming a father. This study was guided by the Consolidated Criteria for Reporting Qualitative Research Checklist.

## Background

The transition to parenthood is described as a major (Seefat-van Teeffelen et al., 2011) and an overwhelming life event (Barimani et al., 2017). A wide range of emotions accompanies the transition to parenthood (Schumacher & Meleis, 1994). It can make fathers more sensitive (Solberg et al., 2023), regardless of becoming a father for the first or the second time (Chin et al., 2010). Parenthood can be highly stressful to fathers, as it is a permanent and demanding transition (Vidaurreta et al., 2022). A healthy transition to parenthood involves a sense of well-being related to comfort with the behaviors required of parents and role achievement (Schumacher & Meleis, 1994). Becoming a parent can evoke negative emotions, which are among the main factors impacting the well-being and mental health of fathers (Baldwin et al., 2018). Despite its importance, little is known about changes in existential dimensions of well-being when becoming a parent, particularly in men (Brandel et al., 2018).

Becoming a father is described as an existential phase when fathers recognize themselves as adults (Åsenhed et al., 2013), which can stimulate personal growth under normative and stressful circumstances, that is, full-term and premature births (Porat-Zyman et al., 2017). As the transition to parenthood is a critical period in a new father’s life, ensuring a smooth transition increases the likelihood that the father will be more involved and engaged in the process (Bakermans-Kranenburg et al., 2019). Previous research has categorized fathers’ transition to parenthood into four phases: beginning a journey, fatherhood in limbo, facing reality, and settling down (Vidaurreta et al., 2022). Beginning a journey starts with the decision to have a baby and represents men’s ambivalence between an active and passive position. Fatherhood in limbo includes dealing with feelings of proximity and remoteness. Facing reality includes coping with their feelings and new situations, while settling down comprises sensing mastery through normalization. The pregnancy phase is the most demanding for fathers, requiring significant psychological reorganization of the self (Genesoni & Tallandini, 2009). The postnatal period is partly about integrating a new social status as a father, which can be challenging, dedicating less focus to their careers (Genesoni & Tallandini, 2009).

Contemporary fathers’ transition to parenthood within a digital societal context both entails and stimulates different conditions compared to pre-digital generations of fathers. *Digital tools*, which refer to smartphones and social media, have become an integral part of life (The Internet Foundation, 2023), with many contemporary fathers using such tools to seek support, guidance, and information (Satir, 2023). This usage, in turn, influences their experiences and roles in parenthood. For example, many fathers use digital means to socialize with friends (The Internet Foundation, 2023) and connect with other parents (Lupton, 2016). Social networks have become a crucial means by which fathers generally prepare for childbirth and parenthood (Bäckström et al., 2021). Although digital tools can be useful, they can fuel anxiety. Digital tools present unparalleled opportunities to expand social connections and better understand and adapt to parenthood, potentially improving the health and well-being of parents (Bäckström et al., 2022). Experiencing well-being includes having the capacity to contribute meaningfully to the world (World Health Organization, 2023). The primary drive of human beings is to make meaning and have, as Frankl (1990) called it, the will for meaning. The essential meaning of the childbirth experience for first-time fathers has been described as alternating between euphoria and anxiety (Premberg et al., 2011).

Swedish citizens’ society strongly emphasizes individuality and secular values, meaning less emphasis on religion and traditional family values (World Value Survey, 2023). Cohabiting, living in the same household in a marriage-like form without being married, is common in Sweden; in 2011, 1.3 million cohabited (Eurostat, 2015).

Fathers’ experiences during the birth of their child can have a lasting impact on them as fathers navigate the postpartum period, with negative childbirth experiences being associated with a higher rate of depression and a lower sense of security (Courtois & Wendland, 2023). Stress related to preserving a work–life balance can stem from fathers envisioning themselves in traditional roles, namely as providers for and supporters of their partners and children (Ghaleiha et al., 2022). The expectation is no longer that mothers should prioritize domestic duties while fathers focus on breadwinning (Genesoni & Tallandini, 2009). Previous research has shown that reduced parental stress in fathers has been linked to sharing parental leave equally (Lidbeck et al., 2018) and better perceptions about the quality of their relationship (Lidbeck & Bernhardsson, 2020).

Research has shown that many first-time fathers receive inadequate support and services from healthcare professionals as they are tailored primarily to women (Høgmo et al., 2021). Fathers often report feeling neglected and lack father-specific support throughout their journey to parenthood (Baldwin et al., 2019; Hodgson et al., 2021), with some claiming that they have experienced inequality from child healthcare (CHC) nurses (Hrybanova et al., 2018). This experience can be attributed to uncertainty on the part of fathers about what to ask for, as well as feelings of exclusion and the desire for family safety and care (Høgmo et al., 2021).

As fathers’ experiences of transition to parenthood are affected by various aspects, it is imperative to gain a comprehensive understanding of their experiences to address what it means to become a father. Such an understanding can be essential for supporting a healthy transition to parenthood. Conducting research describing the experiences of becoming a father is needed to fill the knowledge gap, which can be used by professionals supporting expectant and new parents. This study aims to illuminate the meaning of becoming a father, during the transition to parenthood, involving pregnancy, birth, and the first 2 years of a child’s life.

## Method

This qualitative study applied a phenomenological hermeneutical approach, as described by Lindseth and Norberg (2004). This approach entails data collection through interviews designed to encourage participants to share their experiences in their own words. The study adhered to the Consolidated Criteria for Reporting Qualitative Research (COREQ) Checklist for qualitative research, ensuring rigorous reporting and transparency (Tong et al., 2007).

### Setting

The study was conducted in Sweden from August 2022 to January 2023. In Sweden, pregnant mothers receive standardized care from midwives at midwifery units (from gestational weeks 5–7), a follow-up visit after birth (8–16 weeks), and access to CHC centers for regular check-ups until their child turns 6. Fathers are invited to attend meetings in antenatal care (AC) units and CHC services, which are accessible and free to families. Family centers integrate various activities for families and their children, including AC, CHC, open preschools, and preventive social services.

### Participants and Recruitment

**Figure 1.**
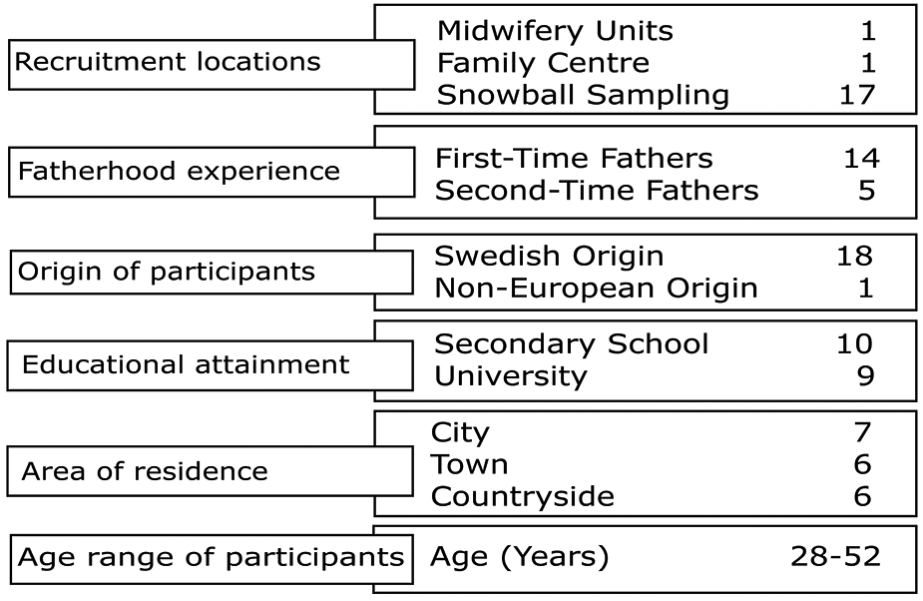
Participants’ characteristics.

Purposive sampling was used to recruit participants (Polit & Tatano Beck, 2020): men who were waiting to become a father (*n* = 5) and men who recently became fathers (*n* = 14) for the first (*n* = 14) or second time (*n* = 5). Participants were recruited in two ways: (a) local recruitment at midwifery units (*n* = 1) and family centers (*n* = 1) in the study area in southwestern Sweden and (b) via snowball sampling (*n* = 17) nationwide in Sweden (Bryman, 2016), which occurred concurrently with the interviews (see Figure 1). Locally recruited participants received information and written and verbal information about the study aim and procedure from the midwives or family center pedagogues. The first author (ÅL) provided written and verbal information to those recruited through snowball sampling.

In total, 22 participants were recruited based on the following criteria: they either had a pregnant partner or their partner had given birth to their child within the past 2 years. Three participants ultimately dropped out of the study, reducing the total number of participants to 19. The fathers were aged between 28 and 52 years. Fathers younger than 18 and those receiving psychiatric treatment were excluded from participation. All participants were involved in a heterosexual relationship and living together with their child’s mother, as cohabiting (*n* = 13) or married (*n* = 6). Additionally, all participants lived in Sweden when the study was conducted. Nearly half of the participants (*n* = 9) finished university, and the rest (*n* = 10) finished secondary school. The participants lived in a city (*n* = 7), town (*n* = 6), or in the countryside (*n* = 6). One father reported a preterm born child. The other father’s children were born without health issues (in a normal birth). The participants were, at the time of the interview, working (*n* = 14), studying (*n* = 1), and on paternal leave (*n* = 4), meaning that their spouse went back to work.

### Data Collection

Data collection involved 19 open-ended interviews with first-time (*n* = 14) and second-time (*n* = 5) fathers, between September 2022 and January 2023. The first author carried out individual interviews (one interview/participant), at various points throughout pregnancy (*n* = 3), from pregnancy week 20 and up to 19 months after childbirth (*n* = 16). The fathers could choose to be interviewed by video, at the family center or during a walk. Of the fathers, 13 preferred to be interviewed via video call, while 3 at the family center, and 3 fathers preferred to be interviewed during a walk. No relationship with participants existed prior to the start of the study, with contact limited to email correspondence for interview scheduling. Before each interview, the first author introduced herself to the participant as a midwife and mother who was interested in his experiences of becoming a father. An interview guide was developed by the first author based on previous research (Gamgam Leanderz et al., 2021). The guide was pilot-tested in one interview, after which the questions were adjusted. Data from the pilot test interview were not included in the subsequent study. To assess the participants’ experiences of becoming a father, the initial open-ended question was “How are you?” followed by “What does it mean for you to become a father?,” “How do you handle being/becoming a father?,” and “What makes you feel well?.” Sub-questions and probing questions were used to encourage reflection (Creswell, 2017), such as “Can you please tell more?” and “Can you please give an example?” with openness to their narratives to achieve a detailed description. The duration of the interviews was 35 to 73 min (51 min on average). It was all audio recorded and transcribed verbatim by the first author, resulting in a total of 240 pages of transcribed text.

### Data Analysis

A phenomenological hermeneutical approach was adopted to analyze the interviews (Lindseth & Norberg, 2004, 2021). The analysis began with an initial data reading and then formulating a naïve understanding. This concept, as described by Ricoeur (1976), refers to the early stages of interpreting text, in which our preunderstanding shapes our initial perceptions of the text.

The structural analysis began by reading the whole text and dividing it into meaning units. These meaning units were not constrained by length so long as they conveyed a single meaning. The meaning units were subsequently condensed and composed concisely and accessibly. The interpretative process involved entering a hermeneutic circle, one in which parts were analyzed to form a new whole, iterating between understanding and explanation, a movement between the story as a whole, which gave meaning to the parts, which in turn gave meaning to the whole story and formed a new whole. The condensed meaning units were repeatedly reviewed concerning their similarities and differences and reorganized into subthemes multiple times. As the analysis progressed, these subthemes were abstracted into broader themes. In this phase, an existential perspective was pursued by asking questions about the experiences of becoming a father as a reflexive exercise (Sigurdson, 2016). The themes were compared to the initial naïve understanding, which was reformulated and re-examined through additional structural analyses. The author’s preunderstandings are grounded in being women and mothers and being from different healthcare disciplines (midwives, occupational therapists, primary health nurses, or intensive care nurses). The preunderstanding has been used, even though bridled. What was put in brackets were the judgments about the objective. This was made to become open to the experience and to the unspoken meaning of the experience (Lindseth & Norberg, 2021). This process was repeated five times over 6 months, with discussions among the co-authors recurring until a consensus was reached and a relevant description of the phenomenon was provided. The naïve understanding guided the structural analysis and ensured its validation (Lindseth & Norberg, 2021). The structural analysis resulted in 2 themes and 10 subthemes.

### Ethical Considerations

Ethical approval was obtained from the Ethical Review Board (Reference number: 2022-00413-01; 2022-02868-02). Before being interviewed, the participants received detailed written and verbal information about the study, including its objective, methods, eligibility criteria, and the participant’s right to withdraw at any time. The participants provided consent to participate in a digital format in accordance with the Declaration of Helsinki (World Medical Association, 2013). To ensure confidentiality, personally identifiable information was removed from all data. Only authorized persons had access to interview responses. The data were stored, secured, and managed in accordance with the General Data Protection Regulation.

## Results

### Naïve Understanding

Becoming a father is an unimaginable life adjustment—their relationship with their partner changes, and a longing for physical closeness remains. The responsibilities associated with parenthood hit them as they realize they are forever connected to their child and have feelings they had not anticipated. Preparations focus on practical tasks, while faith or friends can help ease anxiety. Fathers are devoted to family, prioritizing their child, although personal interests sometimes take a backseat. Solitude and chosen relaxation offer energy and rest. They seek to bond with their child through daily interactions, embracing significant changes and new routines while experiencing self-growth and a fresh perspective on life.

#### Themes and Subthemes

The analysis resulted in two themes: to feel connectedness and to create strategies for the new situation. The themes are further explained in 10 subthemes (Table 1).

**Table 1.** Results for the Themes and Subthemes.

Themes	Subthemes
To feel connectedness	To spend time with partner and adjusting the relationship
	To feel a responsibility
	To feel confident and engaged in the present
	To feel ambivalence
	To be united by boundless love
	To feel social belonging
To create strategies for the new situation	To be flexible
	To manage one’s worries
	To use digital media for support
	To find space and solitude

#### To Feel Connectedness

A feeling of connectedness is essential to the experience of becoming a father. Fathers feel this connectedness when spending time with their partner, adjusting the couple’s relationship, and with the new responsibilities. Fathers feel confident and engaged in the present but likewise feel ambivalence, impacting the feeling of connectedness. They feel connectedness as being united by the boundless love for their child and by feeling social belonging.

##### To Spend Time With Partner and Adjusting the Relationship

From a father’s perspective, adjusting his relationship with his partner involves mutually supporting each other by wanting each other to be well, and by giving and taking in equal measure to strike a healthy balance.

Fathers expressed the need for closeness with their partners, greater focus on each other, and feeling seen in the relationship. They wished to spend more time together with their partners, as doing so permitted more opportunities for physical closeness:

“You have become three [people] full-time, but the fact that you are still trying to create space to be just two can be very important.” (234).

They wanted to feel needed and loved by their partner for who they were. Adjustments to couple relationships involved creating and spending time together.

##### To Feel a Responsibility

To become a father is related to feelings of responsibility, which the fathers experienced as a strange feeling, yet one that was rewarding and exciting. Being responsible for children of their own and being part of a family that they helped create was deeply meaningful, as life was no longer about just themselves:

“The philosophical perspective is that it means everything; in my world, it is the meaning of life, to become a parent, to raise a nice and kind person.” (229)

Central for feelings of responsibility was the fathers’ focus on their child’s well-being before their own, with their child’s needs awakening a strong desire to be close to and care for their child—to be fully present and spend more time with their child. The fathers describe how they sought mutual respect as parents and took responsibility for their relationship by maintaining open communication and reflecting on their feelings and their thoughts. The fathers express the importance of parental leave, as it allows them to take greater responsibility for their relationship with their children and to achieve greater authenticity in their role:

“Many people say that you should make the most of your time with your child. I feel like I am doing it now. The only thing I can regret is that I did not take more parental leave.” (239).

The fathers’ sense of responsibility for their children is a profound experience, one awakened when they fully realize how vulnerable and dependent their child is on them.

##### To Feel Confident and Engaged in the Present

The fathers recognized that inner confidence was fundamental to feeling confident as a father. They described being affected in feeling more confident by previous experiences, such as caring for people and animals. Fathers listened to and compared themselves with other fathers and noted similarities they could use to enhance their sense of security. They discussed how they did what they wanted without listening to other people’s opinions—for example, via social media. They needed to grow into their parental role and become more engaged in the present moment as they learned to be fathers. They realized that there are no shortcuts; it takes time to develop parenting skills:

“It is not possible to acquire the qualities you get as a parent in any other way than just becoming a parent, and it is not possible to teach it to yourself; you have to put in these hours and hours again, all the time you put in creating this encounter, cannot be compared with so much else.” (248)

By being in the present moment, the fathers ensured that their child’s basic needs were met and felt confident in their ability to comfort and care for them. For example, they knew to put away their cell phones to focus on their child. Paying attention to the small things that happen in everyday life, to the minutiae in the here and now, is an effective way to engage in the present without focusing on what came before or is to come.

##### To Feel Ambivalence

The fathers recognized that it can take time to integrate becoming a father within themselves, and that, consequently, the emotional significance of the role can be delayed, making them feel ambivalent. For some, becoming a father can be experienced as an immense and formidable challenge—even a trap, one that isolates and restrains them. It can be exhausting to look after both oneself and one’s child. Some fathers expressed feelings of guilt due to moments when they lacked appropriate emotions and were not happy about having a child. One father described the existence of his child as his choice:

“Still think I am important, and now it is a double effort to look after yourself as well as another person [the child] who did not choose this, without it being something one [the father] has chosen to be brought to the world.” (245)

Becoming a father can be experienced as a burden, and fathers can experience inner conflict when contradictory and ambivalent emotions toward being a father and toward the child emerge and linger. Fathers’ emotions can come from experiencing a duality, missing the life they had, being constrained, and knowing they are supposed to be content.

##### To Be United by Boundless Love

The fathers reflected on how their boundless love for their children emotionally touched them and united them with their children. Realizing how much it means to be a father can only occur when one holds his child in his arms and recognizes that this relationship is eternal. Making eye contact with their child gave the fathers a feeling of exchange and togetherness, both central to feeling united by boundless love. The fathers felt needed and loved when they experienced their child’s affection—when being hugged, for example. Seeing their child happy lit a fire within the fathers and produced a sense of inner joy they had not experienced before:

“A side of love that I could not understand what it meant, how much it would mean, it was probably a feeling that I did not know I had in my body, that I cannot describe.” (247)

The fathers reflected on seeing themselves in their child, their origins, and how they can make an impression on the world through their child. Their love for their child was so strong that the thought of someone taking them made them faint.

##### To Feel Social Belonging

Social belonging for the fathers meant being in contexts where they could receive support from partners, friends, parents, and co-workers. For example, fathers on parental leave can still visit their workplace or friends. They can socialize with friends worldwide via digital media.

The fathers described needing deep relationships and having friends with whom they could share their innermost feelings, even their most ambivalent ones, about becoming a father. Sharing their thoughts and feelings with someone who listened to them validated them as a person. Fathers who believed in God found solace in communicating via prayer:

“Have shared a lot of the feelings I may have had with becoming a father; you have been able to get to the bottom of it and talk about how it feels to become a parent; that is what has been so nice to have had someone to talk to about the good things but also the things that may not be bad but things that are difficult for you.” (245)

On the other hand, superficial contact and small talk were deemed less meaningful by the fathers.

#### To Create Strategies for the New Situation

Central for the meaning of becoming a father was to create strategies for their new situation. They achieve this by being flexible to the circumstances of everyday life, being able to manage their worries, and using digital media for support and finding solitude.

##### To Be Flexible

The fathers use the strategy to be flexible to make their daily life run smoothly. For example, in child-rearing, fathers sometimes have different experiences and duties than their partners and must compromise. Everyday life with their child means being flexible as well as balancing between providing guidance and relying on natural progress:

“With a child, it is a bit like riding a torrent; you just have to adapt to where the torrent is going, try to steer as best you can to avoid the rocks, then you can have as much fun as you can, do not have a set plan, decide exactly where you have to stay, you simply have to rely on nature, and it is quite fun to adapt a little. Otherwise, you follow a strict path.” (248)

The fathers admitted to having high expectations of parenthood but accepted that everyday life was now shared with their children and that they took each day as it came. One way to practice flexibility is by reducing ambitions, for example, house renovations and career goals, and instead seeking to enjoy life without unnecessarily complicating it. Even though long-term plans and goals are intended to provide purpose and guidance in life, achieving harmony and balance between work and leisure is a worthwhile and rewarding pursuit. In this respect, parental leave is crucial, contributing to achieving such a balance.

##### To Manage One’s Worries

Becoming a father was depicted as a shattering life event, one accompanied by many worries and an increased sense of inner vulnerability. This may be attributable to previous experiences of miscarriage and abortion, feelings of anxiety, and panic before an impending birth, or having a child with a disability. The fathers wished to manage their worries by using their time and energy wisely, by focusing on what they could control and not worrying about what they could not—for example, by studying or relying on their faith:

“Prayed a lot, talked a lot with the priest so everything would go well, and luckily it did. I was worried about everything that was involved with parenthood, I got support talk [from the priest], tips and advice, a bit about how to behave, and what would happen, and read a lot on the Internet about childbirth. But, I was not sure that she would survive—anything can happen during childbirth.” (227)

Some fathers may experience fear and worry about the potential fragility of their child or their child’s rapid development. They may also fear being incapable as a parent due to fatigue or decreased energy. One way to manage worries is to change one’s daily routines, although doing so can be upsetting and unsettling, at least initially. The fathers dealt with their worries by attempting to adopt new routines for themselves and their families.

##### To Use Digital Media for Support

The central meaning of becoming a father is to be knowledgeable. The fathers search information and knowledge about being a parent through digital media, which is easily accessible. Fathers receive information that they need to have the skills to judge the level of quality in having a good quality and the contrary. Performing Google searches for relevant information was described as yielding both reliable information as well as conflicting or incorrect answers, making these fathers feel even more insecure:

“[The jungle of information] refers to the amount of pure information available on the Internet. If you type it into Google, you will find many different blogs with very different quality of information. Sometimes, there can be blogs with opinions. There is a variance in quality and truthfulness in what is found.” (246).

The fathers used digital media primarily for support, for following their partner’s pregnancy via apps, and for searching for information about childbirth. Watching birth videos on digital media was described as a way to prepare for an impending birth but as well as a source of increased anxiety. Some fathers felt that podcasts were a source of support for them in becoming parents. Others participated in digital forums. They can participate in digital forums that provide information about the expected development of children at a particular time, which can awaken thoughts about and engage fathers to participate actively in the child’s achieved development:

“Because there are certain stages in growing up that the child does things and in that he can you read about how to help the child with such things, when you read it, then you get a food for thought on how to do, or continue to coordinate it more, so it is great tips in that way, it actually is.” (253)

The fathers mentioned being affected by the influence of social media on their partners. Watching their partner feel pressured by images and ideas circulating online about what it takes to be a perfect parent compelled them to emphasize that being a parent is only part of what is inherent within a person:

“I am not just a father; I am a husband, an official, a friend, I am myself, I am myself in all of this; I think you should remember that you are the whole alphabet, not just A or B.” (249)

The fathers recognized that the transition to parenthood has worked for others throughout history and would thus work for them.

##### To Find Space and Solitude

The meaning of becoming a father includes finding space for solitude and having solitude allowed them to care for themselves better, on their terms, and to spend time on hobbies and interests. For them, occasional solitude made life manageable. Going to the cinema, working out at the gym, listening to music, cooking, watching TV, or gardening are just a few examples of solitary activities. Some fathers mentioned playing video games as a means to find peace in the moment—but no joy in the long run. Solitude was described as necessary for reflecting on current circumstances, processing thoughts, or just relaxing:

“It can be just walking down the dirt road here, for ten minutes, clearing some thoughts, and shaking off the day. It can mean a lot; it can be just lying on the lawn if it is summer and warm and nice.” (217)

The fathers described wanting to rest after work, relax, be productive, develop ideas, and implement projects. Before becoming fathers, they used time to be alone to do what they liked. For example, communing with nature can accelerate recovery from a day at work, while volunteer work is meaningful as it is worthwhile and beneficial for oneself and others. Solitude is an opportunity when spontaneity is restricted, and personal freedom can be constrained, but long-term planning can make room for meeting friends.

## Discussion

This study applied a phenomenological hermeneutical approach to illuminate the meaning of becoming a father, foremost of which is to feel connectedness and to create strategies for the new situation in this life-altering experience. Previous research on the meaning of becoming a mother revealed the great extent to which mothers in a sense of belonging were deeply touched and transformed by motherhood (Gamgam Leanderz et al., 2025), indicating that fathers and mothers are similarly feel connectedness. For fathers to feel connectedness, being physically close to their partner is one aspect. They need closeness to feel seen and want to spend more time together—unlike mothers, who struggle to build relationships with their children and wrestle with thoughts that they are not good enough mothers (Gamgam Leanderz et al., 2025). The relationship quality between partners who are expecting a child plays a crucial role in shaping the father’s level of engagement and involvement, even when they are committed to childcare (Griffith et al., 2023). Such differences in terms of the need for closeness with a partner can, unfortunately, be fertile ground for separation (Gamgam Leanderz et al., 2021). Research by Lidbeck and Bernhardsson (2019) underscored the importance of equal parental leave for both partners, as it has been shown to promote healthier co-parenting dynamics and a better work–life balance for both mothers and fathers.

The results show that the meaning of becoming a father is grounded in creating strategies for the new life situation. Fathers do this by being flexible in everyday life toward their partner, and engaging with their child. They must also manage their worries, trust the process of becoming a father, and accept that they cannot control everything. To support fathers in creating strategies for their new life situation, healthcare professionals in AC and CHC must give fathers equal treatment as mothers, as they are both parents (Björk, 2016). In practice, this would mean giving fathers a place in meetings with healthcare professionals (Björk, 2016). Research has revealed that during individual conversations with non-birthing parents, CHC nurses can help fathers reflect on their living situation to work toward greater equality and apply a family-focused approach (Larsson et al., 2020). Individual conversations tailored to the father’s needs allow for a different type of content, making the father feel essential (Massoudi et al., 2023). This can, in turn, strengthen the father’s ability to create strategies for their new living situation. Also, they can create their space by participating in fatherhood groups led by a male leader, which helps them form social networks (Berlin et al., 2020).

Contemporary fathers navigate their transition to parenthood in a digital society. The result of our study illuminates that fathers use digital media for support in their transition to parenthood. They are driven by the desire to feel knowledgeable about being a parent, but digital media can be a source that can evoke anxiety. Research describes a significant interest among expectant and new fathers in using digital media to prepare to become a father and support their health (Da Costa et al., 2017). Fathers read blogs, perform Google searches, and participate in digital forums. Forums primarily can provide fathers with detailed information about the developmental trajectory of their children, consequently engaging them more actively in their child’s development and ultimately benefiting their health and improving their well-being (World Health Organization, 2007). The fathers in our study described experiencing anxiety from watching birth videos on digital media. Research shows that fathers fearing childbirth cope by being aware of their feelings (Nordin-Remberger et al., 2024). The fathers in our study felt confused or insecure about the different, often contradictory information received through digital media. In Sweden, a full 100% of Swedes aged 16 to 64 years use the Internet (The Internet Foundation, 2024). The ubiquity of the Internet allows parents to be online anywhere and at any time via digital media (Lupton, 2016).

Given that fathers use digital media when becoming fathers, the importance of guidelines for healthcare professionals on how they can modify their support to meet the specific needs of fathers must be highlighted and addressed. Research has shown that support should be included to enable a partner’s participation (Gamgam Leanderz et al., 2021). As the capacity of fathers to participate in preparatory support may be limited during the daytime due to work, digital tools can be used to allow healthcare professionals and fathers to meet online. Midwives and child health nurses can provide parents with physical and digital support to enhance collaboration. Direct communication channels between parents and healthcare professionals can lay the groundwork for enhanced collaboration and co-production in healthcare (Eysenbach, 2008). With digital tools, an environment can be created that encourages parents to become more actively involved and create value in their care (Palumbo, 2017). Expanding digital tools in healthcare can bridge the gap between healthcare professionals and parents (Palumbo, 2017). Healthcare professionals should seek out fathers on digital media to leverage these opportunities and interact with them (Maged, 2013). As Rosa (2021) noted, digitalization has transformed our relationship with the world, providing unprecedented access to almost anyone worldwide. With parents spending an average of several hours daily on digital media (Van Kessel et al., 2022), it is essential to acknowledge its impact on their lives. Our results showed that fathers reflect on and often put their cell phones away to focus on their children. Digital media has changed our environment and our relationships with each other and ourselves (Burr et al., 2020). The fathers in this study found deep meaningfulness in being responsible for children and being part of a family that they had a will to form, and life was no longer about just themselves. The fathers in this study make meaning by creating strategies and have the will for meaning (Frankl, 1959/1992).

### Methodological Considerations

A key strength of this study was that the interviews were conducted with fathers during their transition to parenthood, allowing for the capture of their experiences in real time. The sample included both fathers-to-be and new fathers as well as first- and second-time fathers, providing variability in our findings—this laid the ground for a varied sample and several viewpoints, which were a ground for achieving theoretical saturation. With 19 participants, our study generated a thick description of the fathers’ experiences (Lincoln & Guba, 1985), rich with meaningful accounts and details (Bryman, 2016). This thick description not only enhanced the transferability of the findings but also provided detailed insights into the experience of Swedish fathers in heterosexual relationships where half of them were university educated, from that aspect, a relatively homogeneous group. However, the homogeneity of this group could influence the transferability of the research results to other contexts. Additionally, the use of digital interviewing allowed the fathers to participate from the comfort of their own homes. In contrast, the walking interviews permitted the fathers to let their thoughts wander freely.

To ensure the credibility and rigor of the study, we carefully followed the analytical steps developed by Lindseth and Norberg (2004, 2021). Quotations were used to strengthen the trustworthiness and validity of the findings. Additionally, we maintained awareness of our preconceptions. The first author regularly met with the four co-authors: all women from different healthcare disciplines (midwifery, primary healthcare, intensive care, and occupational therapy) to share personal experiences as parents, strengthening the trustworthiness of the findings.

## Conclusion

Fathers reflect on the meaning of becoming a father as they are deeply affected by the new situation. Their feeling of connectedness to the family is central. Fathers act to create strategies that respond to and are flexible with their specific situation while also ensuring they have time for solitude. They also seek support and guidance through digital media to better manage becoming a father. This knowledge can be used by healthcare professionals when giving support, for example, meeting face-to-face or online. This support can help fathers explore themselves by reflecting on the meaning of becoming a father.

### Clinical Implications

To understand and fully support the meaning fathers give to the experience of becoming a father and navigating their delicate transition to parenthood, midwives and CHC nurses should facilitate reflective conversations about the fathers’ experiences. Building on previous research, healthcare professionals could ask parents questions such as: “How do you understand your role in childbirth?”; “What does it mean to you to become a parent?”; “How do you manage unpredictability?” (Gamgam Leanderz et al., 2021). Midwives and CHC nurses can go online to meet fathers in parental forums and encourage reflective conversations. With this approach, what it means to become a father can be more fully understood and appreciated, facilitating their transition to parenthood.
